# Draft Genome Sequence of *Acetobacter tropicalis* Type Strain NBRC16470, a Producer of Optically Pure d-Glyceric Acid

**DOI:** 10.1128/genomeA.01329-14

**Published:** 2014-12-18

**Authors:** Hideaki Koike, Shun Sato, Tomotake Morita, Tokuma Fukuoka, Hiroshi Habe

**Affiliations:** a Bioproduction Research Institute, National Institute of Advanced Industrial Science and Technology (AIST), Tsukuba, Ibaraki, Japan; b Research Institute for Innovation in Sustainable Chemistry, National Institute of Advanced Industrial Science and Technology (AIST), Tsukuba, Ibaraki, Japan

## Abstract

Here we report the 3.7-Mb draft genome sequence of *Acetobacter tropicalis* NBRC16470^T^, which can produce optically pure d-glyceric acid (d-GA; 99% enantiomeric excess) from raw glycerol feedstock derived from biodiesel fuel production processes.

## GENOME ANNOUNCEMENT

Acetic acid bacteria (*Acetobacteriaceae*) are obligate aerobes that show efficient oxidation of a wide range of substrates, such as alcohols, sugars, sugar acids, and sugar alcohols. These bacteria have numerous membrane-bound dehydrogenases and oxidoreductases to accomplish such oxidative reactions; therefore, energy-consuming transport of substrates into the cell and products out of the cell is not required (1). Among acetic acid bacteria, *Acetobacter* sp. have been used for industrial vinegar production due to their strong ethanol-oxidizing ability, and their complete or draft genome sequences were reported previously (e.g., 2, 3).

We previously reported a biotechnological method for producing optically pure d-glyceric acid (d-GA) with a 99% enantiomeric excess (ee) from glycerol using *Acetobacter tropicalis* NBRC16470^T^. The maximal 101-g/liter·day GA productivity under optimized conditions was achieved using this strain (4, 5). d-GA is a promising compound that likely exerts biological activities, such as d-GA-promoted acceleration of ethanol metabolism in rats (6) and d-GA-promoted proliferation of human dermal fibroblasts (7).

The *A. tropicalis* NBRC 16470^T^ draft genome was generated using the next-generation sequence platform, Illumina MiSeq (Illumina, San Diego, CA). A paired-end DNA library (insert size, ~500 bp) was prepared using an NEBNext Ultra DNA library prep kit for Illumina (New England Biolabs, Ipswich, MA) and sequenced using the MiSeq reagent kit v2. Sequence data were generated, totalling 2.2 M paired-end reads, each 250 bp in length. Genomic sequence assembly using the SOAPdenovo2 assembler (8) generated 453 scaffolds composed of 654 contigs and a 3.7-Mb draft genome sequence at 151-fold coverage from the paired-end library. The length of the longest scaffold was 394,032 bp, and the *N*_50_ length was 97,875 bp with 11 scaffolds. Protein-coding genes were predicted using the Microbial Genome Annotation Pipeline (MiGAP) system (9), which identified a total of 3,347 coding sequences. A total of 42 tRNA-encoding genes and three rRNA-encoding genes were also identified.

Previously, a draft genome sequence of a thermotolerant strain, *Acetobacter tropicalis* SKU1100 (NBRC101654), isolated from fruit in Thailand, was reported (10). However, that of the *A. tropicalis* type strain (NBRC16470) has not yet been analyzed. In *A. tropicalis*, we demonstrated that the membrane-bound alcohol dehydrogenase (mADH) subunit I-encoding gene (*adhA*) was involved in d-GA production from glycerol (11). Another two genes encoding mADH subunits were also identified in the *A. tropicalis* NBRC16470 genome: the cytochrome *c* subunit II and an AdhS-like subunit III (12). Several other membrane-bound dehydrogenases were annotated from the genome information, including membrane-bound aldehyde dehydrogenase and pyrroquinoline quinone-dependent glucose dehydrogenase. The draft genome sequence of *A. tropicalis* NBRC16470 may provide novel molecular information and increase our understanding of the production mechanism of high optical pure d-GA from glycerol.

### Nucleotide sequence accession numbers.

The *A. tropicalis* NBRC16470 genome sequence (accession number BBMU00000000) was deposited as 654 contigs (accession numbers BBMU01000001 to BBMU01000654) and 453 scaffolds (accession numbers DF850035 to DF850487) at DDBJ/EMBL/GenBank. The version described in this study is the first version.

## References

[1] Matsushita K Toyama H Adachi O 1994 Respiratory chains and bioenergetics of acetic acid bacteria. Adv. Microb. Physiol. 36:247–301. 10.1016/S0065-2911(08)60181-2. 7942316

[2] Illeghems K De Vuyst L Weckx S 2013 Complete genome sequence and comparative analysis of *Acetobacter pasteurianus* 386B, a strain well-adapted to the cocoa bean fermentation ecosystem. BMC Genomics 14:526. 10.1186/1471-2164-14-526. 23902333 PMC3751514

[3] Hung JE Mill CP Clifton SW Magrini V Bhide K Francois JA Ransome AE Fulton L Thimmapuram J Wilson RK Kappock TJ 2014 Draft genome sequence of *Acetobacter aceti* strain 1023, a vinegar factory isolate. Genome Announc. 2(3):e00550-14. 10.1128/genomeA.00550-14. 24903876 PMC4047455

[4] Habe H Fukuoka T Kitamoto D Sakaki K 2009 Biotransformation of glycerol to d-glyceric acid by *Acetobacter tropicalis*. Appl. Microbiol. Biotechnol. 81:1033–1039. 10.1007/s00253-008-1737-2. 18853153

[5] Habe H Shimada Y Yakushi T Hattori H Ano Y Fukuoka T Kitamoto D Itagaki M Watanabe K Yanagishita H Matsushita K Sakaki K 2009 Microbial production of glyceric acid, an organic acid that can be mass produced from glycerol. Appl. Environ. Microbiol. 75:7760–7766. 10.1128/AEM.01535-09. 19837846 PMC2794115

[6] Eriksson CJ Saarenmaa TP Bykov IL Heino PU 2007 Acceleration of ethanol and acetaldehyde oxidation by d-glycerate in rats. Metabolism 56:895–898. 10.1016/j.metabol.2007.01.019. 17570248

[7] Sato S Kitamoto D Habe H 2014 In vitro evaluation of glyceric acid and its glucosyl derivative, α-glucosylglyceric acid, as cell proliferation inducers and protective solutes. Biosci. Biotechnol. Biochem. 78:1183–1186. 10.1080/09168451.2014.885823. 25229854

[8] Luo R Liu B Xie Y Li Z Huang W Yuan J He G Chen Y Pan Q Liu Y Tang J Wu G Zhang H Shi Y Liu Y Yu C Wang B Lu Y Han C Cheung DW Yiu SM Peng S Xiaoqian Z Liu G Liao X Li Y Yang H Wang J Lam TW Wang J 2012 SOAPdenovo2: an empirically improved memory-efficient short-read *de novo* assembler. GigaScience 1:18. 10.1186/2047-217X-1-18. 23587118 PMC3626529

[9] Sugawara H Ohyama A Mori H Kurokawa K 2009 Microbial genome annotation pipeline (MiGAP) for diverse users, abstr S-001, p 1–2 20th Int Conf Genome Informatics, Kanagawa, Japan.

[10] Matsutani M Hirakawa H Nishikura M Soemphol W Ali IA Yakushi T Matsushita K 2011 Increased number of arginine-based salt bridges contributes to the thermotolerance of thermotolerant acetic acid bacteria, *Acetobacter tropicalis* SKU1100. Biochem. Biophys. Res. Commun. 409:120–124. 10.1016/j.bbrc.2011.04.126. 21554859

[11] Habe H Sato S Fukuoka T Kitamoto D Yakushi T Matsushita K Sakaki K 2011 Membrane-bound alcohol dehydrogenase is essential for glyceric acid production in *Acetobacter tropicalis*. J. Oleo Sci. 60:489–494. 10.5650/jos.60.489. 21852749

[12] Yakushi T Matsushita K 2010 Alcohol dehydrogenase of acetic acid bacteria: structure, mode of action, and applications in biotechnology. Appl. Microbiol. Biotechnol. 86:1257–1265. 10.1007/s00253-010-2529-z. 20306188

